# Utility of monocyte distribution width in the differential diagnosis between simple and complicated diverticulitis: a retrospective cohort study

**DOI:** 10.1186/s12876-023-02736-0

**Published:** 2023-03-28

**Authors:** Chang-Yuan Chang, Tai-Yi Hsu, Guan-Yi He, Hong-Mo Shih, Shih-Hao Wu, Fen-Wei Huang, Pei-Chun Chen, Wen-Chen Tsai

**Affiliations:** 1 College of Medicine, China Medical University, Taichung, Taiwan; 2 Department of Emergency Medicine, China Medical University Hospital, Taichung, Taiwan; 3 Department of Public Health, China Medical University, Taichung, Taiwan; 4 Department of Dermatology, National Taiwan University Hospital Yunlin Branch, Yunlin, Taiwan; 5 College of Medicine, National Taiwan University, Taipei, Taiwan; 6 Department of Exercise Health Science, National Taiwan University of Sport, Taichung, Taiwan; 7 Department of Medical Research, China Medical University Hospital, China Medical University, Taichung, Taiwan; 8 Department of Health Services Administration, China Medical University, No. 100, Sec. 1, Jingmao Rd., Beitun Dist., Taichung City, 406040 Taiwan

**Keywords:** Colonic diverticulitis, Monocyte distribution width, Neutrophil-to-lymphocyte ratio, Platelet-to-lymphocyte ratio

## Abstract

**Background:**

Colonic diverticulitis is a leading cause of abdominal pain. The monocyte distribution width (MDW) is a novel inflammatory biomarker with prognostic significance for coronavirus disease and pancreatitis; however, no study has assessed its correlation with the severity of colonic diverticulitis.

**Methods:**

This single-center retrospective cohort study included patients older than 18 years who presented to the emergency department between November 1, 2020, and May 31, 2021, and received a diagnosis of acute colonic diverticulitis after abdominal computed tomography. The characteristics and laboratory parameters of patients with simple versus complicated diverticulitis were compared. The significance of categorical data was assessed using the chi-square or Fisher’s exact test. The Mann–Whitney U test was used for continuous variables. Multivariable regression analysis was performed to identify predictors of complicated colonic diverticulitis. Receiver operator characteristic (ROC) curves were used to test the efficacy of inflammatory biomarkers in distinguishing simple from complicated cases.

**Results:**

Of the 160 patients enrolled, 21 (13.125%) had complicated diverticulitis. Although right-sided was more prevalent than left-sided colonic diverticulitis (70% versus 30%), complicated diverticulitis was more common in those with left-sided colonic diverticulitis (61.905%, *p* = 0.001). Age, white blood cell (WBC) count, neutrophil count, C-reactive protein (CRP) level, neutrophil-to-lymphocyte ratio (NLR), platelet-to-lymphocyte ratio (PLR), and MDW were significantly higher in the complicated diverticulitis group (*p* < 0.05). Logistic regression analysis indicated that the left-sided location and the MDW were significant and independent predictors of complicated diverticulitis. The area under the ROC curve (AUC) was as follows: MDW, 0.870 (95% confidence interval [CI], 0.784–0.956); CRP, 0.800 (95% CI, 0.707–0.892); NLR, 0.724 (95% CI, 0.616–0.832); PLR, 0.662 (95% CI, 0.525–0.798); and WBC, 0.679 (95% CI, 0.563–0.795). When the MDW cutoff was 20.38, the sensitivity and specificity were maximized to 90.5% and 80.6%, respectively.

**Conclusions:**

A large MDW was a significant and independent predictor of complicated diverticulitis. The optimal cutoff value for MDW is 20.38 as it exhibits maximum sensitivity and specificity for distinguishing between simple and complicated diverticulitis The MDW may aid in planning antibiotic therapy for patients with colonic diverticulitis in the emergency department.

## Background

A diverticulum is a herniation through a weak site of the bowel wall that produces a small outpouching [1]. When the diverticular wall is eroded by increased intraluminal pressure or inspissated food particles, diverticulitis may occur [2]. Colonic diverticulitis is one of the most common causes of abdominal pain and lower gastrointestinal bleeding in the emergency department (ED). The prevalence of colonic diverticulitis is increasing not only in Western countries but also in Asian countries [3]. It is predicted that approximately 50% of individuals aged 60 years or older have diverticulosis, whereas by the age of 80 years, this percentage is predicted to be approximately 70% [4]. Of those who developed diverticulosis, 10%–25% experienced an acute episode of diverticulitis [5]. Colonic diverticulitis is usually diagnosed in the ED through intravenous (IV) contrast computed tomography, which is the modality of choice for the diagnosis and staging of colonic diverticulitis with a sensitivity of 94% and a specificity of 99% [6]. Simple acute diverticulitis is a self-limiting and mild disease. It is defined as localized inflammation without any abscess or perforation [7]. The clinical symptoms include lower abdominal pain, fever, constipation, and diarrhea. Outpatient treatment is required for patients who have simple non-septic diverticulitis, are immunocompetent, and can tolerate oral intake. However, approximately 15% of diverticulitis cases have been reported to be complicated forms and were manifested with abscess, stricture, obstruction, fistulae to adjacent organs, or perforation [8–10]. As a consequence of bacterial translocation, fecal contamination, or phlegmon development, complicated diverticulitis may present with severe abdominal pain, bloating, dehydration, and signs of sepsis [11]. On physical examination, patients may exhibit peritonitis with rebound tenderness and guarding. Patients with complicated diverticulitis must receive treatment specific to their complications. The current therapeutic options for diverticulitis vary with disease severity, which can be determined based on clinical, radiological, and laboratory findings. When diffuse peritonitis is suspected given the findings of a physical examination, emergency surgery may be required even if imaging shows that the abscess is localized [12]. Therefore, early assessment of the severity of complicated diverticulitis and adequate resuscitation are important.

The monocyte distribution width (MDW) is a novel hematological parameter assessed as part of the complete blood count (CBC) with the differential count. It helps in determining the size distribution of circulating monocytes, which are the first immune cells to respond to pathogenic organisms [13]. In a multicenter international European study, the MDW in combination with the white blood cell (WBC) count was suggested to be a novel screening test for the early detection of sepsis in the ED [14]. Comparison of diagnostic performance according to the Sepsis-3 criteria revealed that the MDW was not inferior to the C-reactive protein (CRP) or procalcitonin level in terms of area under the receiver operator characteristic (ROC) curve (AUC) values [15]. Few studies have focused on the efficacy of the MDW in diagnosing diseases other than sepsis. To our knowledge, the MDW has been used for the detection of the novel coronavirus disease (COVID-19) [16–18] and pancreatitis [19]. However, there is a lack of evidence on the efficacy of the MDW in early prediction of the severity of other diseases.

In this retrospective cohort study, we aimed to investigate whether the MDW data preceding CT assessment is helpful in differentiating simple from complicated colonic diverticulitis in an ED.

## Methods

### Study design and setting

This retrospective cohort study was conducted at a university-affiliated medical center receiving approximately 150,000 ED visits annually. The study was approved by the Hospital Ethics Committee on Human Research. The study protocol was reviewed and qualified as exempt from the requirement to acquire informed consent.

### Patient selection

Patients older than 18 years who presented to our ED between November 1, 2020, and May 31, 2021, and who received a diagnosis of acute colonic diverticulitis after abdominal CT was performed were included in this study. All the enrolled patients received the indicated blood examinations. Patients who had any other concomitant active inflammation or infection, were receiving any antibiotic course, had a final pathological diagnosis other than colonic diverticulitis, or had incomplete medical records or laboratory data were excluded from the study. Data were retrieved from the institutional electronic medical chart of the ED.

### Methods and measurements

The collected variables included patient demographics and laboratory data. Blood tests were obtained at the same time inserting the IV line within an hour after the treating physicians’ examination of patients at the ED. Blood samples were acquired before antibiotic treatment and IV contrast CT scan. The patients’ age; sex; and comorbidities—such as diabetes mellitus, hypertension, ischemic heart disease, heart failure, liver cirrhosis, cholelithiasis, rheumatological disease, asthma, chronic obstructive pulmonary disease, chronic renal insufficiency, urolithiasis, cerebrovascular disease, and existing cancer—were recorded. Moreover, the body temperature upon triage and laboratory findings (including WBC, neutrophil, lymphocyte, and platelet counts; MDW; and hemoglobin, CRP, creatinine, alanine aminotransferase, blood sugar, sodium, and potassium levels) were recorded.

All the patients’ admission diagnoses coded as diverticulitis were reviewed, then IV contrast CT images were confirmed by two ED physicians and revalidated with the radiologists’ formal final reports whether there was a perforation, abscess, or fistula formation. Based on the IV contrast CT findings, the patients were divided into simple colonic diverticulitis and complicated colonic diverticulitis groups. The radiographic features of simple diverticulitis on CT are enhancement of the colonic wall with segmental thickening and pericolic fat stranding, often disproportionately prominent compared to the amount of bowel wall thickening. As for the complicated types, accumulation with fluid or/with gas suggested abscess formation. And extravasation of gas and fluid into the pelvis and peritoneal cavity are the characteristics of diverticular perforation [20, 21].

The MDW was measured by the UniCel DxH 900 analyzer (Beckman Coulter, Brea, CA, USA) from K3EDTA vacutainer tubes within 2 h of collection, as recommended by the manufacturer. The analyzer uses volume, conductivity, and scatter properties of leukocytes technology to characterize and separate WBCs into 5 different groups (neutrophils, eosinophils, monocytes, lymphocytes, and basophils). The system furthermore calculates the means and standard deviations of these groups’ cell morphometric parameters [22]. The manufacturer of the hematology analyzer did not offer the unit for MDW, as previously reported [23, 24].

### Statistical analysis

Descriptive statistics were used to compare variables—baseline demographics, laboratory test results, and inflammatory biomarker measurements—between the two groups. Categorical variables are expressed as proportions, and continuous variables are expressed as medians with interquartile ranges (IQRs, quartile 1 through quartile 3). Univariate analysis was performed using the chi-square or Fisher’s exact test for categorical variables and the Mann–Whitney U test for continuous variables in order to identify predictors of complicated colonic diverticulitis. Variables with *p*-values < 0.10 in the univariate analysis were then subjected to backward stepwise logistic regression analysis. ROC curves were also used to assess the performance of inflammatory biomarkers— including the WBC count, neutrophil-to-lymphocyte ratio (NLR), platelet-to-lymphocyte ratio (PLR), MDW, and CRP level—in order to distinguish simple from complicated colonic diverticulitis. Youden’s indices were calculated on ROC curves to find the best discriminatory cut-off values [25]. Test characteristics of MDW in terms of sensitivity, specificity, positive predictive value, and negative predictive value along with their 95% CIs for the optimal cut-off value were also computed. A *p*-value ≤ 0.05 was considered statistically significant. All data were analyzed using SAS (version 9.1; SAS Institute, Cary, NC, USA). Post-hoc power of the study was estimated using G*Power software (version 3.1.9.7; Heinrich Heine University Düsseldorf, Germany) with the α error probability set at 0.05.

This study was approved by the Institutional Review Board of the Ethics Committee of China Medical University and Hospital (CMUH110-REC3-106). The requirement of informed consent from the patients was waived by the Ethics Committee of China Medical University and Hospital.

## Results

**Fig. 1:**
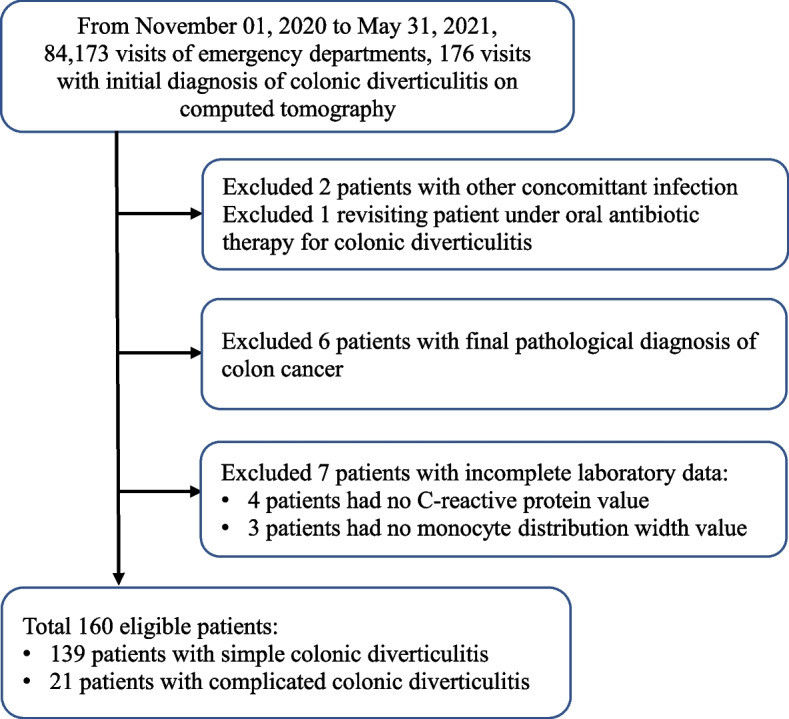
The flowchart of the enrolled study patients

From November 1, 2020, to May 31, 2021, 84,173 ED visits were recorded. A total of 176 visits were retrieved from the institutional electronic medical chart during the study period. Of the 16 patients who were excluded from the study, 2 had other concomitant infections, 1 was a repeat patient who had revisited the ED and was taking oral antibiotics for colonic diverticulitis, 6 had an initial ambiguous diagnosis of colonic diverticulitis on CT scans but a final pathological diagnosis of colon cancer and not diverticulitis, 4 had no CRP data, and 3 had no MDW data because their monocyte count was less than 100/μL. Thus, a total of 160 patients were enrolled in the study: 139 in the simple colonic diverticulitis group and 21 in the complicated colonic diverticulitis group (Fig. 1).

Overall, the number of male patients was higher than that of female patients (*n* = 90/160, 56.25%). Most patients had right-sided colonic diverticulitis (*n* = 112, 70%): 14 in the cecum (8.75%), 91 in the ascending colon (56.875%), and 7 in the transverse colon (4.375%). Seventeen patients (10.63%) had other diverticula in different segments of the colon. Recurrent colonic diverticulitis was noted in 27 patients (16.88%). Hypertension was the most prevalent comorbidity (*n* = 30, 18.75%). Comparison of the variables for the two groups revealed that the patients with complicated colonic diverticulitis were older, with a median age of 58 years (IQR: 41–72 years). Conversely, the patients with simple colonic diverticulitis had a median age of 44 years (IQR: 30–59 years; *p* = 0.008). Complicated colonic diverticulitis was more frequently detected in the left colon than in the right colon (61.905% versus 38.095%). By contrast, simple colonic diverticulitis was more prevalent in the right colon than in the left colon (74.180% versus 25.180%). The location of colonic diverticulitis significantly differed between the two groups (*p* = 0.001). However, no significant difference was noted in comorbidities between the two groups. Laboratory values—WBC (*p* = 0.008) and neutrophil (*p* = 0.003) counts, MDW (*p* < 0.001), and CRP level (*p* < 0.001)—were significantly higher in the complicated colonic diverticulitis group. By contrast, lymphocyte count (*p* = 0.001) and sodium level (*p* < 0.001) were significantly higher in the simple colonic diverticulitis group (Table 1).

**Table 1:** Distributions of baseline variables by colonic diverticulitis group

Variables	Total	Simple Diverticulitis	Complicated Diverticulitis	*p*-value
Patients, n (%)	160 (100)	139 (86.875)	21 (13.125)	
Female sex, n (%)	70 (43.75)	62 (44.604)	8 (38.095)	0.642
Age in years, median (IQR)	45 (31.25–60)	44 (30–59)	58 (41–72)	0.008*
Body mass index	23.821 (20.701–26.892)	23.875 (20.700–26.892)	23.624 (20.695–26.388)	0.950
Body Temperature (℃), median (IQR)	36.8 (36.5–37.375)	36.8 (36.5–37.3)	37.2 (36.45–37.5)	0.273
Recurrent diverticulitis, n (%)	27 (16.875)	21 (15.108)	6 (28.571)	0.129
Multiple locations of diverticula, n (%)	17 (10.625)	15 (10.791)	2 (9.524)	1.000
Location of diverticulitis, n (%)				0.001*
Right colon:	112 (70)	104 (74.820)	8 (38.095)	
*Cecum*	*14 (8.750)*	*14 (10.072)*	*0 (0)*	
*Ascending colon*	*91 (56.875)*	*85 (61.151)*	*6 (28.571)*	
*Transverse colon*	*7 (4.375)*	*5 (3.597)*	*2 (9.524)*	
Left colon:	48 (30)	35 (25.180)	13 (61.905)	
*Descending colon*	*24 (15)*	*19 (13.669)*	*5 (23.810)*	
*Sigmoid colon*	*24 (15)*	*16 (11.511)*	*8 (38.095)*	
Comorbidities, n (%)				
Diabetes mellitus	10 (6.25)	9 (6.475)	1 (4.762)	1.000
Hypertension	30 (18.75)	25 (17.986)	5 (23.810)	0.551
Ischemic heart disease	8 (5)	7 (5.036)	1 (4.762)	1.000
Heart failure	2 (1.25)	2 (1.439)	0 (0)	1.000
Liver cirrhosis	1 (0.625)	1 (0.719)	0 (0)	1.000
Cholelithiasis	10 (6.25)	9 (6.475)	1 (4.762)	1.000
Rheumatologic disease	2 (1.25)	1 (0.719)	1 (4.762)	0.246
Asthma / COPD	2 (1.25)	1 (0.719)	1 (4.762)	0.246
Chronic renal insufficiency	4 (2.5)	3 (2.158)	1 (4.762)	0.434
Urolithiasis	10 (6.25)	7 (5.036)	3 (14.286)	0.127
Cerebrovascular disease	1 (0.625)	1 (0.719)	0 (0)	1.000
Existing cancer	4 (2.5)	4 (2.878)	0 (0)	1.000
Laboratory Data, median (IQR)				
WBC × 10^9 /L	11.15 (8.7–13.775)	10.9 (8.6–13.6)	13.3 (11.0–15.45)	0.008*
Neutrophils (%)	76.75 (69.825–82)	75.2 (68.8–81.4)	80.1 (76.8–86.3)	0.003*
Lymphocytes (%)	14.2 (9.7–20.05)	14.9 (10.5–20.9)	9.5 (5.9–14.25)	0.001*
MDW	18.78 (17.44–20.6475)	18.56 (17.21–19.77)	22.66 (20.655–24.465)	< 0.001*
Platelets × 10^9 /L	243 (196–286)	244 (197–286)	227 (188.5–310)	0.893
NLR	5.357 (3.478–8.363)	5.040 (3.305–7.524)	8.372 (5.431–15.057)	0.001*
PLR	152.383 (121.295–207.622)	148.457 (120.351–196.479)	206.860 (141.476–366.163)	0.017*
CRP (mg/dL)	3.835 (0.9675–9.06)	3.46 (0.67–6.08)	12.14 (5.235–15.42)	< 0.001*
Alanine aminotransferase (U/L)	18.5 (12–26.75)	18 (12–26)	19 (12–34.5)	0.750
Creatinine (mg/dL)	0.8 (0.6525–0.9575)	0.79 (0.66–0.95)	0.86 (0.61–1.135)	0.471
Sodium (mmol/L)	139 (137–140)	139 (138–140)	137 (136–138)	< 0.001*
Potassium (mmol/L)	3.7 (3.5–3.9)	3.7 (3.6–3.9)	3.7 (3.4–3.9)	0.251
Glucose (mg/dL)	104.5 (94–123)	105 (94–123)	103 (92–135)	0.860

*IQR* Interquartile range, *COPD* Chronic obstructive pulmonary disease, *WBC* White blood count, *MDW* Monocyte distribution width, *NLR* Neutrophil to lymphocyte ratio, *PLR* Platelet to lymphocyte ratio, *CRP* C-reactive protein, *ALT* Alanine aminotransferase

^*^ *P* < 0.05

Univariate and multivariable binary logistic regression analyses (Table 2) revealed that left-sided location and the MDW were the only two variables that were significant predictors of complicated colonic diverticulitis after adjusting for the other variables. The adjusted odds ratio (OR) of complicated colonic diverticulitis was 5.197 (95% confidence interval [CI], 1.651–16.359; *p* = 0.005) for left-sided location and 1.552 (95% CI, 1.290–1.867; *p* < 0.001) for the MDW.

**Table 2:** Univariate and multivariable binary logistic regression analyses showing independent predictors of complicated diverticulitis

Variables	Unadjusted OR (95% CI)	*p-*value	Adjusted OR (95% CI)	*p-*value
Age	1.037 (1.009–1.066)	0.010*	–	–
Left colon	4.829 (1.848–12.616)	0.001*	5.197 (1.651–16.359)	0.005*
WBC	1.189 (1.051–1.346)	0.006*	–	–
CRP	1.166 (1.083–1.256)	< 0.001*	–	–
MDW	1.584 (1.304–1.923)	< 0.001*	1.552 (1.290–1.867)	< 0.001*
NLR	1.063 (1.003–1.127)	0.038*	–	–
PLR	1.005 (1.002–1.008)	0.004*	–	–
Sodium	0.718 (0.594–0.867)	0.001*	–	–

Variables with *p* < 0.10 in Table 1 were selected into logistic regression. Neutrophils and lymphocytes were eliminated because of their multicollinearity with NLR and PLR

The value of the Hosmer–Lemeshow test for the multivariable logistic regression is 0.827 (> 0.05), which indicates that the model’s estimate fits the data at an acceptable level

*WBC* White blood count, *CRP* C-reactive protein, *MDW* Monocyte distribution width, *NLR* Neutrophil-to-lymphocyte ratio, *PLR* Platelet-to-lymphocyte ratio, *OR* Odds ratio, *CI* Confidence interval

^*^ *p* < 0.05

**Fig. 2:**
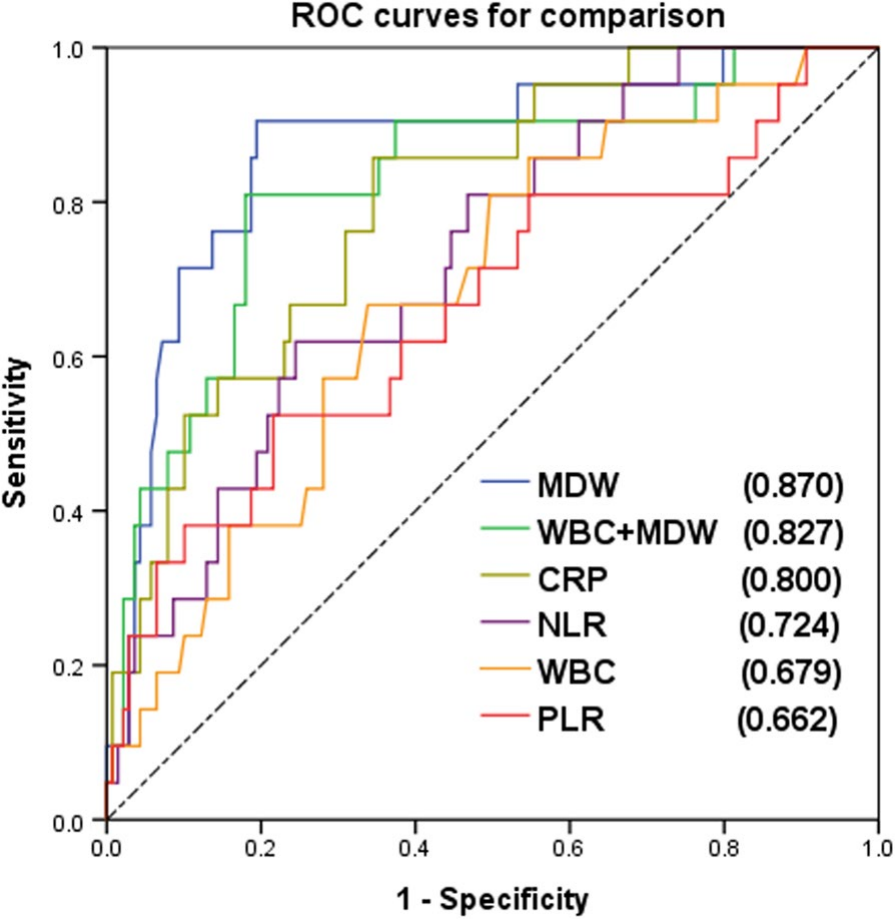
Receiver Operator Characteristics (ROC) analysis of MDW and other inflammatory biomarkers (MDW = monocyte distribution width; CRP = C-reactive protein; NLR = neutrophil lymphocyte ratio; WBC = white blood cell; PLR = platelet to lymphocyte ratio.)

**Fig. 3:**
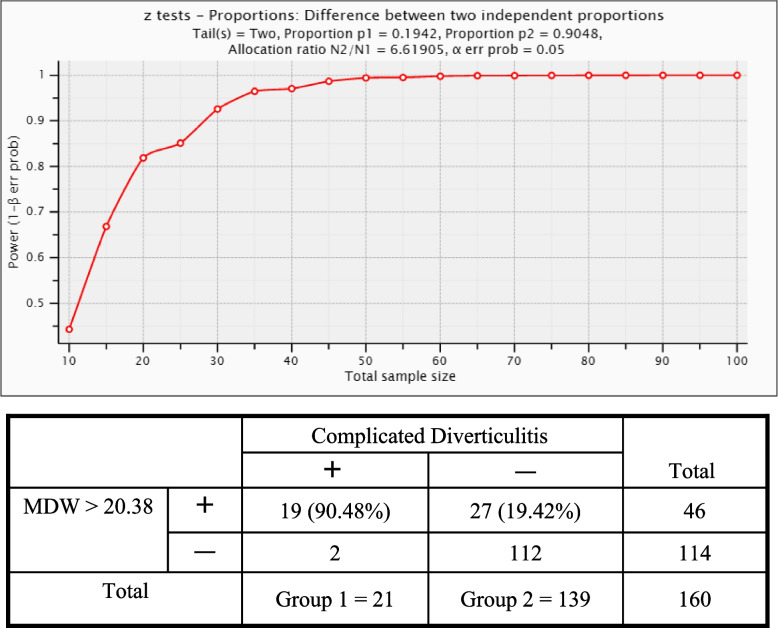
Sample size calculation with G*Power for monocyte distribution width value of 20.38

Further evaluation through ROC analysis was performed to determine the diagnostic value of the MDW for complicated colonic diverticulitis. The AUC values were as follows: MDW, 0.870 (95% CI, 0.784–0.956); WBC + MDW, 0.827 (95% CI, 0.725–0.928); CRP, 0.800 (95% CI, 0.707–0.892); NLR, 0.724 (95% CI, 0.616–0.832); WBC, 0.679 (95% CI, 0.563–0.795); and PLR, 0.662 (95% CI, 0.525–0.798) (Fig. 2 and Table 3). The largest AUC value was that for the MDW among all the inflammatory biomarkers for diagnosing patients with complicated colonic diverticulitis. When the MDW cutoff was 20.38, the sensitivity and specificity were maximized to 90.5% and 80.6%, respectively, with a low positive predictive value of 41.3% but a high negative predictive value of 98.3% (Table 4). A post-hoc power analysis was performed through G*power, which revealed that the sample size was adequate to achieve 100% power (1–β) when the MDW cut-off value was 20.38. The power exceeded 80% when the total sample size was greater than 20 (Fig. 3).

**Table 3:** Area under the curve (AUC) values of the inflammatory biomarkers for complicated diverticulitis

Inflammatory biomarkers	AUC	Standard error	*p-*value	95% Confidence interval
MDW	0.870	0.045	< 0.001	0.784–0.956
WBC + MDW	0.827	0.052	< 0.001	0.725–0.928
CRP	0.800	0.048	< 0.001	0.707–0.892
NLR	0.724	0.059	0.001	0.616–0.832
WBC	0.679	0.060	0.008	0.563–0.795
PLR	0.662	0.071	0.017	0.525–0.798

*MDW* Monocyte distribution width, *WBC* White blood cell, *CRP* C-reactive protein, *NLR* Neutrophil lymphocyte ratio, *PLR* Platelet to lymphocyte ratio

**Table 4:** The test characteristics of monocyte distribution width for complicated diverticulitis

Cut-off value	Sensitivity (95% CI)	Specificity (95% CI)	Positive predictive value (95% CI)	Negative predictive value (95% CI)
20.38	0.905 (0.696–0.988)	0.806 (0.730–0.868)	0.413 (0.328–0.504)	0.983 (0.937–0.995)

*CI* Confidence interval

## Discussion

From the results of the study, it was concluded that the increase in age, WBC count, neutrophil count, CRP level, NLR, PLR, and MDW were related to the severity of colonic diverticulitis.

The gold standard diagnostic tool for acute diverticulitis is CT, in which complications can also be visualized. However, the schedule of CT at an ED may be delayed because of the high number of patients. The optimal use of CT for patients in whom complicated diverticulitis is suspected should be based on clinical and laboratory findings to minimize treatment costs and radiation hazards [26]. Therefore, recognizing the risk factors of complicated diverticulitis and providing the right treatment before CT imaging are crucial.

Some findings of the present study are in accordance with previous findings. In particular, right-sided diverticulitis was more prevalent than left-sided diverticulitis (70% versus 30%); this finding is compatible with reports from other Asian countries [27–29]. However, a higher number of patients with complicated diverticulitis had left-sided diverticulitis than right-sided diverticulitis (61.905% versus 38.095%); this finding is similar to the findings of previous Japanese and Korean studies [3, 27]. We also found that patients with complicated diverticulitis were older than those with simple diverticulitis (median age, 58 versus 44 years); this finding is also compatible with previous findings. For instance, in a Japanese retrospective multicenter study involving 1,112 patients, although right-sided colonic diverticulitis was more prevalent among the study population (70.1%), left-sided colonic diverticulitis was significantly more common among elderly patients (61.0%) [30]. Right-sided diverticulitis differs from left-sided diverticulitis in many respects. While right-sided diverticulitis is usually congenital and solitary [31, 32], left-sided diverticulitis is usually associated with secondary causes, including dietary factors, constipation, increased colonic pressure, defecation habits, and an irritable bowel. Consequently, left-sided diverticulitis more commonly occurs in older patients [33].

In our study, the WBC and neutrophil count, MDW, and CRP levels were higher in the complicated colonic diverticulitis group (*p* < 0.05, Table 1); however, only the MDW was found to have a statistically significant association with complicated diverticulitis in the multivariable binary logistic regression analysis (*p* < 0.001, Table 2). The WBC count and CRP level are the most common indicators of the severity of intra-abdominal inflammation in the ED. A higher WBC count or CRP level usually indicates a higher level of inflammation. Several studies [26, 34–36] have attempted to calculate the optimal threshold for the WBC count and CRP level in distinguishing complicated diverticulitis from simple diverticulitis; however, so far, no consensus has been reached.

In addition to the WBC count and CRP level, two easily accessible hemogram-derived parameters, namely the NLR and PLR, have been used to predict complicated diverticulitis [37–39]. One study reported that the NLR could predict the need for surgical intervention more accurately than the CRP level and WBC count [37]. Palacios Huatuco et al. recently found the NLR cutoff of 4.2 to be the best diagnostic approach, with a sensitivity of 80% and specificity of 64%, for detecting complicated diverticulitis [38]. Mari et al. found that the PLR had lower diagnostic accuracy than the NLR (AUC values, 0.67%, and 0.75%, respectively) [39].

Circulating neutrophils and monocytes are the first response to pathogenic organisms. The MDW is a parameter that describes the size distribution of circulating monocytes. Several studies have reported that the MDW can be used for the early diagnosis of sepsis in the ED [13, 14, 40, 41]. Similarly, Şenlikci et al. found that the MDW can be used to differentiate mild pancreatitis from nonmild pancreatitis [19]. However, little known is about the efficacy of the MDW in detecting acute complicated diverticulitis. In our cohort, the MDW cutoff of 20.38 had a sensitivity of 90.5% and a specificity of up to 80.6%. Moreover, it had the largest AUC value (0.870) for the diagnosis of acute complicated diverticulitis. The AUC value of the MDW for complicated diverticulitis was higher than those of other inflammatory biomarkers—CRP (0.800), NLR (0.724), WBC (0.679), and PLR (0.662; Table 3 and Fig. 2).

The diagnostic accuracy of the MDW for complicated diverticulitis noted in our study was comparable with that of procalcitonin. In a previous study, the AUC of procalcitonin for complicated diverticulitis was 0.867, with a sensitivity of 81% and specificity of 91% [42]. However, procalcitonin is not routinely used as a biomarker in EDs. In Taiwan, the national health insurance reimbursement price for procalcitonin tests is 1,000 New Taiwan dollars (NT$) [43], which is approximately four times the price for CBC determination (NT$270, including differential WBC count and MDW) [44, 45]. Therefore, procalcitonin testing is preserved as an auxiliary test for patients with ambiguous diagnoses of sepsis or bacterial infection, which cannot be verified on the basis of the WBC count, NLR, or CRP level.

In our study, the MDW was the only inflammatory biomarker that was found to be a significant predictor of complicated colonic diverticulitis after adjusting for other covariables in multivariable binary logistic regression analysis (*p* < 0.001, Table 2). In previous studies, the MDW was found to have some advantages over other biomarkers. In particular, the MDW can be easily measured from the CBC through a blood test in the ED. In addition, the results are obtained faster than those of a biochemistry panel. Use of the MDW has been reported to improve both the clinical and economic outcomes of patients with sepsis in the ED, with the estimated time to antibiotic administration being reduced from 3.98 h to 2.07 h and US$3,460 being saved per hospitalization (US$23,466 versus US$26,926) [46]. By using a combination of the MDW and advanced imaging (CT), ED physicians will be able to diagnose complicated diverticulitis more accurately and in a timely manner, to initiate antibiotic therapy, and to convince surgeons regarding early intervention. Recent guidelines have recommended avoiding the use of antibiotics for otherwise healthy patients with simple diverticulitis [47]. The high negative predictive value (98.3%) of MDW could enhance physicians’ diagnosis and decision-making. Patients with colonic diverticulitis and normal MDW values are unlikely to be complicated. Hence, an early and accurate diagnosis of simple diverticulitis by using the MDW will help reduce the use of antibiotics.

### Limitations

To our knowledge, this is the first study to evaluate the utility of the MDW for diagnosing colonic diverticulitis in the ED. However, our study has some limitations. First, the MDW cannot be measured when the peripheral blood sample for a patient has a monocyte count < 100/μL. In our study, three patients' MDW data were unavailable; these patients had simple diverticulitis. Second, because this was a retrospective study, medical records were not designed for research purposes and did not contain all parameters of interest to the investigators. For instance, the procalcitonin level was not measured for comparison with the MDW. Third, our classification of colonic diverticulitis was based on CT findings. CT has an accuracy of 98% in diagnosing acute diverticulitis; thus, misdiagnosis may occur in 2% of cases [48]. Nevertheless, abdominal CT imaging is still considered the gold standard for diagnosing acute diverticulitis and its complications [49]. Finally, this was a single-center study conducted in only one ED in East Asia; therefore, our finding that diverticulitis was more prevalent in the right colon may not be generalizable to all EDs and other populations. Further prospective studies with larger numbers of patients from multiple centers are needed to more accurately assess the role of the MDW in differentiating simple from complicated colonic diverticulitis.

## Conclusions

Our study revealed that acute colonic diverticulitis was more prevalent in the right colon than in the left colon in Taiwanese patients. Patients with complicated diverticulitis were significantly older and predominantly had left-sided diverticulitis. In addition, a large MDW was found to be a significant and independent predictor of complicated diverticulitis preceding CT assessment in the ED. The optimal cutoff value for MDW is 20.38 as it exhibits maximum sensitivity and specificity for distinguishing between simple and complicated diverticulitis. The MDW may aid in initiating early antibiotic therapy for patients with complicated diverticulitis and in decreasing antibiotic use in patients with simple diverticulitis.

## Data Availability

The datasets generated and analyzed during the current study are not publicly available due to the non-disclosure agreement in IRB restrictions. However, they are available on reasonable request. Please contact Dr. Tai-Yi Hsu for details.

## Abbreviations

AUC: Area under the ROC curve
CBC: Complete blood count
CI: Confidence interval
CT: Computed tomography
ED: Emergency department
IV: Intravenous
IQR: Interquartile range
MDW: Monocyte distribution width
NLR: Neutrophil-to-lymphocyte ratio
OR: Odds ratio
PLR: Platelet-to-lymphocyte ratio
ROC: Receiver operator characteristic
WBC: White blood cell
